# Personalized medicine for reconstruction of critical-size bone defects – a translational approach with customizable vascularized bone tissue

**DOI:** 10.1038/s41536-021-00158-8

**Published:** 2021-08-19

**Authors:** Annika Kengelbach-Weigand, Carolina Thielen, Tobias Bäuerle, Rebekka Götzl, Thomas Gerber, Carolin Körner, Justus P. Beier, Raymund E. Horch, Anja M. Boos

**Affiliations:** 1 Department of Plastic and Hand Surgery and Laboratory for Tissue Engineering and Regenerative Medicine, University Hospital of Erlangen, Friedrich-Alexander-University Erlangen-Nürnberg (FAU), Erlangen, Germany; 2 Institute of Radiology, Preclinical Imaging Platform Erlangen (PIPE), University Hospital Erlangen, Friedrich-Alexander-University Erlangen-Nürnberg (FAU), Erlangen, Germany; 3 Institute of Physics, University of Rostock, Rostock, Germany; 4 Department of Materials Science and Engineering, Institute of Science and Technology of Metals, Friedrich-Alexander-University of Erlangen-Nürnberg (FAU), Erlangen, Germany; 5 Present Address: Department of Plastic Surgery, Hand Surgery, Burn Center, University Hospital RWTH Aachen, Aachen, Germany

**Keywords:** Preclinical research, Translational research

## Abstract

Tissue engineering principles allow the generation of functional tissues for biomedical applications. Reconstruction of large-scale bone defects with tissue-engineered bone has still not entered the clinical routine. In the present study, a bone substitute in combination with mesenchymal stem cells (MSC) and endothelial progenitor cells (EPC) with or without growth factors BMP-2 and VEGF-A was prevascularized by an arteriovenous (AV) loop and transplanted into a critical-size tibia defect in the sheep model. With 3D imaging and immunohistochemistry, we could show that this approach is a feasible and simple alternative to the current clinical therapeutic option. This study serves as proof of concept for using large-scale transplantable, vascularized, and customizable bone, generated in a living organism for the reconstruction of load-bearing bone defects, individually tailored to the patient’s needs. With this approach in personalized medicine for the reconstruction of critical-size bone defects, regeneration of parts of the human body will become possible in the near future.

## Introduction

Therapeutic options for bone defects that cannot heal spontaneously, the so-called critical-size bone defects, are still limited and often associated with a great social burden. For a long time, the gold standard has remained the transplantation of vascularized autologous tissue from an unharmed area of the body, such as the fibula, scapula, or iliac crest^1–3^. Patients suffer from donor site morbidities, long hospital stays, or impaired functions of damaged tissue. From the clinical point of view, there is an urgent need to refine present therapeutic options in order to improve patient care. We have reached a point where current technologies and scientific approaches offer a multitude of encouraging possibilities to adapt bone fracture treatment. However, preclinical data are limited and further proof-of-principle studies are necessary to prepare for the translation of experimental approaches into clinical practice thoroughly.

With bone tissue engineering techniques, researchers developed the possibility to generate living bone tissue in the laboratory adapted to individual patients’ requirements in a highly precise way (reviewed in ref. ^4^). However, when bone defects exceed a certain size, or in the case of impaired vascularization, the tissue-engineered constructs should be characterized by an autonomous blood vessel supply for nutrient and oxygen delivery to guarantee the survival of the transplant and rapid healing of the bone defect (reviewed in ref. ^5^).

There are different options to generate bone replacement with functional tubular structures. Microchannels as precursors for blood vessels can be integrated into materials, e.g., by using sacrificial templates such as sodium alginate^6^ that can be seeded afterward with endothelial cells. Using laser-assisted bioprinting, vascular networks consisting of endothelial cells can be created directly within tissue replacement materials^7^. Vascularization may be further supported by the addition of endothelial cells and pericytes that self-assemble into perfusable vascular tubes after implantation^8^ or by the incorporation of vascular growth factors into matrices. Another approach is the in vivo prevascularization of scaffolds. After implantation into a highly perfused tissue, e.g., subcutaneous or muscular pockets, vessels can grow from the outside into the center of the material until complete vascularization is achieved. To ensure immediate supply of the replacement material after transplantation, tubes within the scaffold have to be interconnected and anastomosis to blood vessels of the recipient site should be possible. Most in vitro prevascularization techniques reach their limits here, because of insufficient vascularization. In the case of in vivo prevascularization in the human body, vascularized materials have to be transplanted as, for example, free hybrid tissue-engineered bone/native autologous muscle—flaps coming along with additional donor site morbidities for the conventional free muscle flap needed as a vascular carrier^9^. A further possibility is the integration of a defined vascular axis for prevascularization, which can be used as a supplying vessel for the transplant before and after transplantation^10^. This technique, using the body as the bioreactor, is known as the arteriovenous (AV) loop or bundle model^11^. The AV loop model has already been successfully used for the vascularization of different matrices for bone tissue engineering. Recently, we were able to demonstrate rapid and complete vascularization of a clinically approved bone substitute in clinically relevant dimensions in a large animal model^12^.

In preparation for clinical translation, we have further developed the AV loop approach in the present study as an innovative therapeutic option for critical-size bone defects. The clinically approved nanocrystalline NanoBone^®^ block, which already showed successful results in our previous studies^12,13^ was seeded with autologous endothelial progenitor cells (EPC) and mesenchymal stem cells (MSC) and either prevascularized in the AV loop or directly transplanted into a critical-size tibia defect in sheep. Growth factors were added to support both angiogenesis and bone regeneration. In the control group, the gold-standard autologous tissue from the iliac crest was implanted. This study should serve as a proof of concept in order to implement this promising approach in the near future in bone defect treatment in the concept of personalized medicine.

## Results

### Isolation and characterization of EPC and MSC

**Fig. 1:**
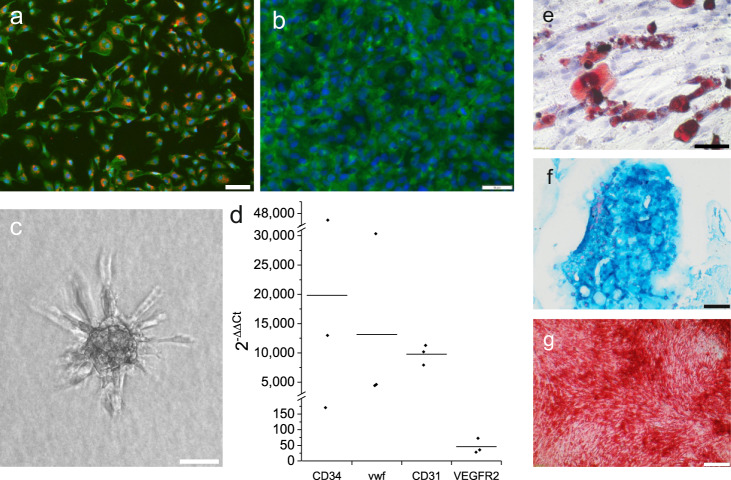
Characterization of sheep EPC and MSC. **a** EPC after uptake of acetylated LDL-DiI (red) and lectin staining (green). Counterstaining with DAPI (blue). Scale bar 50 µm. **b** CD34 immunofluorescence staining (green) of EPC. Counterstaining with DAPI (blue). Scale bar 50 µm. **c** Sprouting of EPC spheroids after 48 h incubation in a fibrin gel. Scale bar 50 µm. **d** mRNA expression of EPC compared to MSC (set to 1). **e** Oil Red O staining of MSC after adipogenic differentiation. Scale bar 100 µm. **f** Alcian Blue staining of MSC after chondrogenic differentiation. Scale bar 200 µm. **g** Alizarin Red staining of MSC after osteogenic differentiation. Scale bar 200 µm.

From all sheep, EPC and MSC could successfully be isolated and expanded. Cells were used in passages 4–6 for implantation. EPC showed typical markers of endothelial cells such as uptake of acetylated LDL (Fig. 1a). They stained positively for lectin and CD34 (Fig. 1a, b). Typical sprouting of cell spheroids could be seen in a 3D hydrogel after 48 h (Fig. 1c). Comparing MSC and EPC mRNA expression (*n* = 3), levels of endothelial cell markers such as CD31, CD34, VEGFR2, and vWF were highly elevated in EPC (Fig. 1d). mRNA expression varied between individual sheep, but in all sheep, a remarkably higher expression of EPC markers in comparison to MSC could be found. MSC were successfully differentiated into adipogenic (Fig. 1e), chondrogenic (Fig. 1f), and osteogenic (Fig. 1g) cell lineages demonstrating their multipotency. Expression of characteristic stem cell markers was shown in the previous studies^14^.

### Surgical procedure

The transplantation of the prevascularized bone-AV-loop construct was successful in all sheep. In spite of scar tissue at the chamber implantation site, the pedicle vessels could be microsurgically exposed and prepared for transplantation. The custom-made tibia-plate-titanium-chamber osteosynthesis showed a perfect fit to the tibia and bone defect and led to a stable fixation of the leg. Microanastomoses showed patency in the Doppler probe. Directly after clamp removal, the prevascularized bone-AV-loop construct showed punctiform bleeding as a sign of sufficient perfusion.

All sheep survived the surgeries well. In some sheep, minor complications like swelling or postoperative bleeding of the surgical wound were observed. No major complication that led to reoperations or exclusion from the study occurred. Postoperative X-ray of the tibia demonstrated a good plate fixation and defect stabilization in all sheep. During the observation period calcification could be observed in the bone defect. In some sheep, 8–12 weeks after the bone defect surgery the skin over the proximal end of the osteosynthesis plate became rather thin and sometimes showed defects measuring 2–5 mm in diameter without any signs of infection. Wounds were treated with conservative wound management and could be treated like that until explantation. From group 3, one sheep had to be excluded due to limited EPC yield in the peripheral blood samples.

From the first postoperative day after the bone defect surgery, sheep were allowed to move freely in the stable. Immobilization of the bone defect, the correct location of the osteosynthesis plate and screws was ensured by the cast and checked regularly by X-ray. After about 6–8 weeks sheep began to completely load the operated leg. The patency of the loop vessels was checked by Doppler sonography once a week. Throughout the complete implantation time a positive arterial and venous signal could be detected in the pedicle and loop vessels in all sheep.

### Bone formation and degradation of bone substitute

**Fig. 2:**
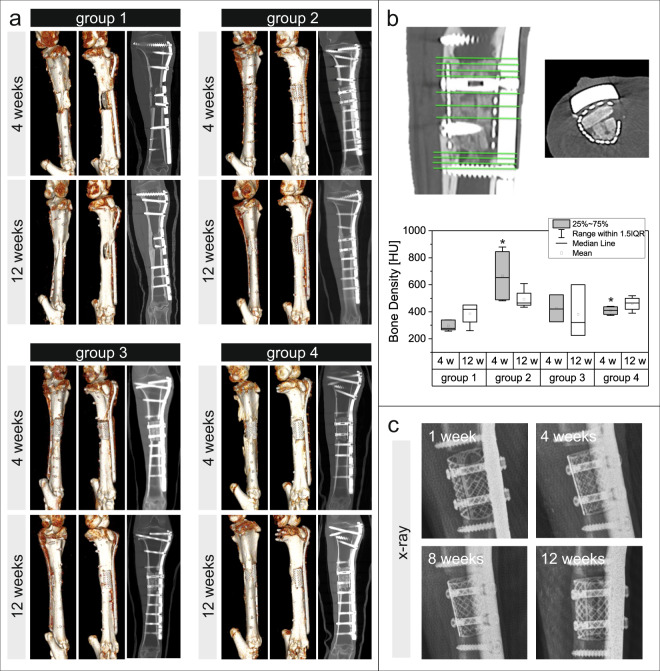
3D imaging and X-ray of bone defects. **a** Pictures show the 3D reconstruction of CT analyses of the complete tibia as volume reconstruction and coronal sections 4 and 12 weeks after implantation. **b** Bone density was measured in 11 different planes distributed over the entire area of the defect. In the axial section bone substitute blocks could clearly be observed within the chamber. Box plot: y-axis shows the bone density in Houndsfield units, x-axis depicts the experimental groups after 4 weeks (gray) and 12 weeks (white). **c** Representative X-ray images of one sheep 1, 4, 8, and 12 weeks after implantation. *p < 0.05 compared to control group 1 (Kruskal–Wallis test/Mann–Whitney *U*-test).

Using CT analyses, the density of defect areas was measured in 11 different planes distributed over the entire area of the bone defect after 4 and 12 weeks (Fig. 2a, b). For technical reasons, at the 12-week time point, only four out of five animals from group 1 could be scanned. In all animals initial bone bridging of the defect could be visualized in CT and X-rays over time (Fig. 2a, c). Comparing the 4-week measurements, a significantly higher density was measured in groups 2 (*p* = 0.014) and 4 (*p* = 0.014) than in the control group 1. The highest density was observed in group 2. In groups 2 and 3 values decreased over time, while there was an increase in group 1 from 4 to 12 weeks.

**Fig. 3:**
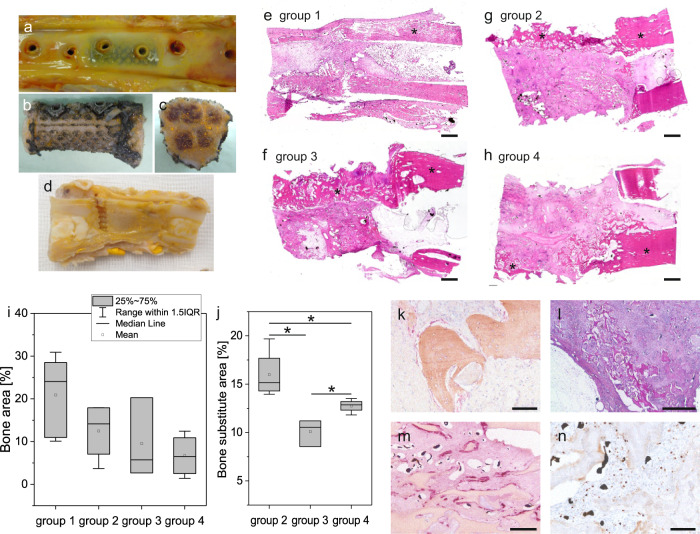
Histological and immunohistological analysis of the newly formed bone area and degradation of the bone substitute. **a**–**d** Macroscopical images of the bone construct. **a** The chamber was completely integrated into the tibia and covered with connective tissue (representative picture of group 2). **b** Bone defect area after removal of the implantation chamber. **c** Cross section of the bone defect area (b/c representative picture of group 3). **d** Longitudinal section of the bone defect area (representative picture of group 1). **e**–**h** HE staining of histological slides of the bone defect area. Intact tibia on the right side of each slide (* = bone tissue). Scale bar 2 mm. **i** Quantification of the bone area and **j** quantification of bone substitute area in percentage (y-axis) of the whole region of interest among different experimental groups (x-axis). **k** Collagen type I (brown)/alkaline phosphatase (pink) double staining (representative picture of group 3). Scale bar 50 µm. **l** PAS staining (representative picture of group 3). Scale bar 1 mm. **m** TRAP staining (pink) for detection of osteoclast-like cells (representative picture of group 2). Scale bar 200 µm. **n** Ki67 immunohistological staining of proliferative, Ki67-positive (brown) cells (representative picture of group 4). Scale bar 50 µm. *p < 0.05 (Kruskal–Wallis Test/Mann–Whitney *U*-test).

After an implantation time of 12 weeks and euthanasia of the animals, the amputated operated legs of the sheep were perfused with the radiopaque contrast agent Microfil^®^. Macroscopically, in all animals the bone substitute within the chamber and the autologous bone in group 1, respectively, were completely integrated into the defect area and covered with connective tissue without any signs of infection (Fig. 3a). The constructs were carefully explanted, always making sure that a small part of the tibia was left at the proximal and distal site of the defect. The titanium chamber was removed (Fig. 3b) and the construct was divided into four pieces (Fig. 3c, d) by cutting/sawing them along their longitudinal axis (Fig. 3d), and then transversally directly in the middle of the constructs (Fig. 3c).

In HE staining (Fig. 3e–h), the edges of the original tibia defect could be visualized, making it easy to identify the ROI for quantification and to measure the newly formed bone and the bone substitute area. In all groups, initial bone bridging of the bone defect could be clearly seen. The diameter of the newly formed bone was always smaller than that of the original tibia cortical bone. The highest percentage of bone tissue was measured in the control group (mean 20.9%). There was no significant difference between the groups. The bone areas of groups 2, 3, and 4 were in the range 6.7–12.5% (mean values), but with high standard deviations (Fig. 3i). Comparing all groups, the bone substitute area was lower in group 3, with a statistical significance of *p* = 0.034, than in groups 2 and group 4. In group 4, significantly less bone substitute could be detected than in group 2 (*p* = 0.021) (Fig. 3j).

Using further (immuno)histological stainings, it could be clearly demonstrated that newly formed bone showed typical expression of bone tissue such as collagen type I and alkaline phosphatase (AP) (Fig. 3k). With PAS staining the typical matrix change of the bone substitute NanoBone^®^ was visible in all regions where the bone substitute was surrounded by newly formed tissue (Fig. 3l). The surface of the NanoBone^®^ particles was always covered by large multinucleated cells. With TRAP staining, it could be shown that these cells are osteoclast-like TRAP-positive cells (Fig. 3m), responsible for the degradation of the bone substitute. All experimental groups showed a high amount of proliferative, Ki67-positive cells (Fig. 3n).

### Vascularization

Vascularization of the bone defect area was measured in 3D using in vivo angio-CT and micro-CT after explantation as well as with histological quantification methods.

**Fig. 4:**
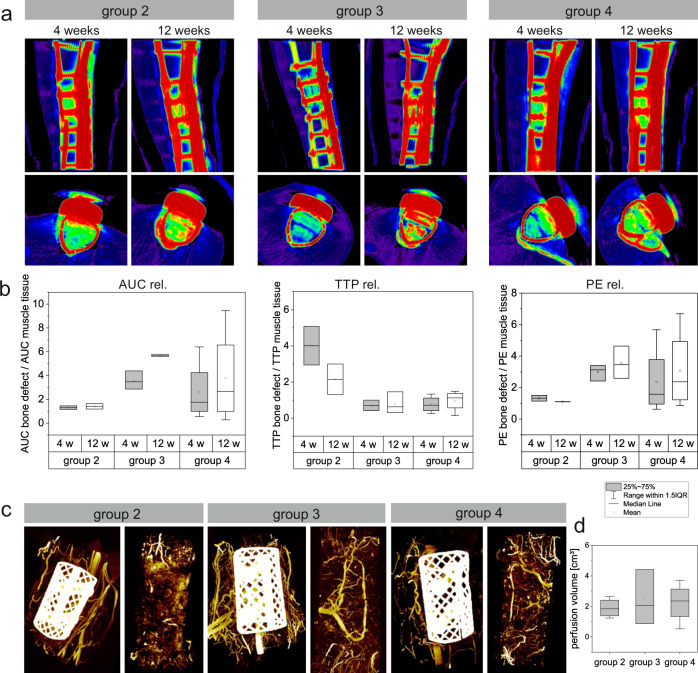
Quantification of vascularization with computed tomography angiography and micro-CT. **a**, **b** Results of computed tomography angiography after 4 and 12 weeks. **a** In experimental groups 2, 3, and 4, an increase in perfused volume could be clearly shown. **b** Quantification of perfused volume in the bone defect area was measured relatively compared to muscle tissue in each animal. Graphs show relative values (y-axis) of the area under the curve (AUC), time to peak (TTP), and peak enhancement (PE) in different experimental groups after 4 and 12 weeks (x-axis). **c**, **d** Micro-CT of bone defects. **c** Bone defects were perfused with the radiopaque contrast agent Microfil^®^. Group 2, 3, and 4: Left picture defect with implantation chamber, right picture after removal of implantation chamber. **d** Quantification of perfusion volume (y-axis) in different experimental groups (x-axis).

In angio-CT analysis, values of groups 2, 3, and 4 were included and not compared to the control group 1 because of special measuring settings due to the titanium chamber. In groups 2 and 3, only two out of three or four animals, respectively, could be measured due to technical problems. Therefore these results were not statistically interpreted. In all animals, it could be clearly demonstrated that the vascularization increased over time from the 4-week to the 12-week measurement time point (Fig. 4a).

Measurements of the area under the curve (AUC), time to peak (TTP), and peak enhancement (PE) were always performed relative to the vascularization in muscle tissue to ensure a high degree of reliability. The AUC, a marker of the signal intensity over time and therefore of how much contrast agent could be measured within the defect area over time, was highest in group 3, while the lowest values were found in group 2. TTP, showing the time until reaching the maximum intensity and therefore a marker of how fast the maximum amount of contrast agent could be measured after the first detection in the defect area, was in the same range in groups 3 and 4, but much higher in group 2. PE, as a marker of the maximum signal intensity, was highest in group 3 and lowest in group 2 (Fig. 4b).

Vascularization was further quantified three-dimensionally using micro-CT after perfusion of the bone defect with the radiopaque contrast agent Microfil^®^ (Fig. 4c). For technical reasons, only the groups with an AV loop (groups 2, 3, and 4) were included in our analysis. In groups 2, 3, and 4, a large amount of perfused vessels could be clearly seen around the chamber and also directly in the defect area. In groups 3 and 4, the AV loop was clearly visible. In group 3, values had a broad distribution, but mean values showed a similar perfused vessel volume in the defect area in all groups without any significant difference (Fig. 4d).

**Fig. 5:**
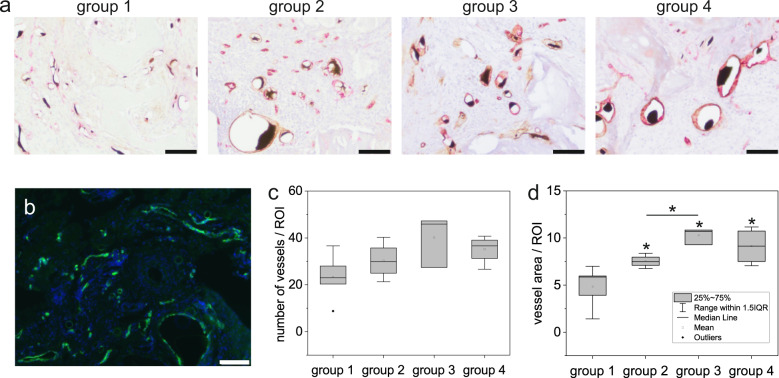
Histological quantification of vascularization. **a** Double-staining α-SMA (brown) and vWF (red). **b** CD31 immunofluorescence staining of bone defects (representative image of group 1). **c** Number of vessels and **d** vessel area were calculated per ROI (y-axis) in the different experimental groups (x-axis). Scale bars 100 µm. *p < 0.05 (Kruskal–Wallis test/Mann–Whitney *U*-test).

Histological slides were immunohistochemically double-stained with α-SMA and vWF (Fig. 5a), and further with CD31 (Fig. 5b). In all groups, a large amount of vascularization could be observed within the entire area of the bone defect. Quantification of the number of vessels and vessel area per ROI revealed the highest values in group 3 (Fig. 5c, d). The vessel area was significantly larger in group 2 (*p* = 0.027), group 3 (*p* = 0.025), and group 4 (*p* = 0.014) compared to the control group 1 and further in group 3 compared to group 2 (*p* = 0.034).

## Discussion

The term “tissue engineering” was first introduced in the late 1980s at a conference entitled “Tissue Engineering” by the National Science Foundation dealing mainly with the manipulation and assembling of living tissues with materials^15^. Currently, tissue engineering is understood as an approach to the generation of functional replacement tissue by combining scaffolds and living cells. Owing to the growing importance of replacing damaged or lost tissue, due to accident survival, the aging of society, and tumor diseases, great attention is being given to this modern strategy. However, even after a couple of decades, tissue-engineered constructs for the reconstruction of large bone defects have not entered the clinical routine. It was the aim of the present study to develop a feasible approach from a clinical perspective to implementing bone tissue engineering strategies in clinical routine.

A lack of vascularization is considered to be one of the main obstacles to translating tissue engineering principles into the clinical scenario^16^. In the case of smaller defects, healthy patients and sufficient vascularization in the defect area, bone healing can be achieved with nonvascularized grafts^17^. In a retrospective case series of mandibular defect reconstructions, it is emphasized that tissue-engineered constructs represent an optimal alternative to autografts even for large defects, but only if there is an adequate vascular supply in the defect area surrounding the tissue-engineered transplant^18^. There are various methods described for introducing vascularization within clinically applied tissue-engineered constructs. Bone substitutes can be combined with vascularized free flap transfer to ensure an immediate supply of nutrients and oxygen in the case of impaired vascularization in the defect area. Melville et al. used a radial forearm flap with a bone allograft supplemented with BMP and bone marrow aspirate for the reconstruction of the maxillary alveolar ridge and could achieve complete healing^19^. Bone substitutes can be implanted within a muscle pouch for prevascularization. After vascularization they are transferred together with the muscle and its supplying vessels into the defect site^9^. Wiltfang et al. prefabricated a bone substitute in combination with BMP-2, cancellous bone chips, and enriched bone marrow aspirate in the gastrocolic omentum^20^. After 3 months the vascularized tissue-engineered construct was transplanted into a large mandibular defect and the supporting vessels microsurgically anastomosed to the local vessels. The transplant remained in situ several months after transplantation and ongoing metabolic activity and bone formation could be seen^21^.

Within our study, we used a similar, but less invasive approach with the aim of inducing minimal donor site morbidity, relying on a subcutaneous surgery and harvesting of a superficial vascular axis without sacrificing any other additional tissue of the patient, e.g., omentum majus or latissimus dorsi muscle, etc., an AV loop was created subcutaneously for axial vascularization. For this approach, it is highly important that the surgeon has experience in microvascular surgery. We consider the possible thrombosis of the AV loop as the biggest risk of this technique and recommend applying anticoagulants after the creation of the AV loop and transplantation of the construct into the defect area. In previous studies, we were able to demonstrate the feasibility of this method for induction of complete vascularization of bone constructs after 12 weeks by combining the intrinsic vascularization by the AV loop vessels and the extrinsic vascularization by the surrounding vessels using a perforated chamber^12^. We transplanted the prevascularized constructs after 4 weeks because at this time point the loop anastomosis was stable and vascularization and bone formation were in the beginning stages without advanced scarring and fibrosis at the prevascularization site. Transplantation is another critical and difficult step of this technique. It is of utmost importance that the AV loop is transplanted and anastomosed to the local vessels with extreme care. In all animals, the AV loop constructs showed clear signs of sufficient perfusion and neoangiogenesis at the time of explantation and after transplantation into the defect area. Wang et al. used a similar approach in a femur defect rat model, but without prior vascularization. The femoral vascular bundle was directly implanted into the scaffold in the defect site leading to a higher degree of bone and vessel formation compared to defects without vascularization^22^. Likewise, Vidal et al. performed a pilot study in sheep metacarpal bone defect, implanting a vascular pedicle from the defect surroundings directly into a 3D-printed bone substitute for supporting vascularization and bone formation^23^. Recently, we were able to demonstrate the successful implementation of in situ tissue engineering in two patients by using a tissue-engineered construct and a vascular axis from the defect surroundings^24^. However, all these techniques are based on sufficient vascularization and the availability of a vascular axis at the defect site, which is probably not the case in infected or irradiated defect areas. By contrast, the bone substitute within the present study was prevascularized in an unharmed area of the sheep’s body to simulate the clinical setting with impaired vascularization at the defect site such as in the case of infections, scarring, or after irradiation or extensive injury. However, one has to notice that patients’ behavior after vascularized transplantation is crucial for the success of this approach. Recently, Warnke et al. established successfully a bone tissue engineering technique in the large animal model which finally was translated into clinical application. A customized mandible replacement was vascularized in the latissimus dorsi muscle and subsequently transplanted into the defect site. Good success was achieved during the first months after transplantation with newly formed bone at the defect site. However, the patient showed poor compliance, e.g., smoking cigarettes, which finally led to necrosis and infection^25^.

To further support neoangiogenesis, VEGF-A was added to the bone substitute in group 2 (without AV loop) and group 3 (with AV loop). VEGF-A is frequently used alone or in combination with BMP-2 for bone tissue engineering. It leads to initial vasculogenesis and can recruit and differentiate MSC into the endothelial cell lineage leading to an improved vascularization^26^. In the AV loop groups 3 and 4, a higher degree of vascularization was observed in the angio-CT than in group 2 without AV loop prevascularization. Experimental imaging of large animals is associated with an extensive effort. For technical reasons, we could not collect angio-CT data from all our animals. Therefore the results of angio-CT could not be analyzed statistically due to there being only a small number of animals in angio-CT measurements.

However, histologically, we were able to demonstrate a significantly larger vessel area in all experimental groups compared to the control group. Further, the AV loop growth factor group (group 3) showed a significantly larger vessel area than group 2 without an AV loop. Including a group with the bone substitute but without VEGF-A and BMP-2 within this study could be an interesting option for comparison with group 4 with an AV loop, but without growth factors. Compared to group 2 there was a clear trend in group 4 for a higher degree of vascularization and further a better bone substitute remodeling. From the clinical point of view, this group 4 could be the most interesting one, because no addition of artificial growth factors with possible related side effects is necessary. Besides supporting neovascularization, VEGF-A has been shown to be also involved in osteogenesis: Feng et al. transfected MSC with VEGF-A and showed that not only vessel density, but also ossification, was significantly enhanced in a rabbit femur defect model compared to the implantation of control nontransfected MSC^27^. Most probably, a higher degree of perfusion supports angiogenesis and osteogenesis and subsequently bone defect healing^28^.

In all our experimental groups we soaked the scaffold with autologous MSC and EPC with and without adding the growth factors BMP-2 and VEGF-A. In our previous studies with the sheep model, an increased amount of bone formation could be observed when combining MSC and BMP-2^13,29^. MSC are approved for clinical application if processed according to Good Manufacturing Practice (GMP). They are well-known for induction of bone formation and are either applied directly or, by using bone marrow as a source of MSC, for bone tissue engineering in patients with successful results^17,20,30–32^. EPC are known to support both osteogenesis and vascularization. Rozen et al. showed that the application of 2 × 10^7^ autologous EPC into the bone defect site of sheep led to a significantly higher degree of bone formation, most probably through stimulation of angiogenesis and vasculogenesis^33^. EPC secrete proangiogenic growth factors such as VEGF, hepatocyte growth factor, granulocyte colony-stimulating factor, and granulocyte-macrophage colony-stimulating factor^34^. They also contribute directly to vascularization through direct incorporation into blood vessels^35^. The combination of the two cell types is well-known to foster bone defect healing^36^ (reviewed in ref. ^37^).

To further foster the positive effect of cell implantations for bone formation, cells can also be injected some weeks after defect surgery into the defect site since oxygen and nutrient supply are rather low at the time point of surgery most probably leading to poor survival of implanted cells. Recently, Berner et al. showed successfully that this technique led to better bone regeneration in a critical-sized tibial defect in the sheep compared to scaffolds that were preseeded with MSC or implanted without MSC^38^.

Radiodensity in CT is generally described by a quantitative scale with Hounsfield units (HU) for the expression of CT numbers in a standardized form. Density in the CT was measured at different levels within the tissue-engineered construct. Interestingly, in the nonvascularized group 2, we observed the highest values of about 600–800 HU and the autologous bone group 1 achieved around half of that (about 300–400 HU, representing “dense cortical-spongy bone”^39^), while the other groups, 3 and 4, with AV loops were in between. It must be borne in mind that the bone substitute also has a certain density, which can lead to high values in the CT measurement. Therefore, we consider the AV loop as supportive for the remodeling of the bone substitute. This observation is further confirmed by the histological quantification: the highest bone substitute area was measured in the nonvascularized group 2 and the lowest in the AV loop group with growth factors (group 3). Combining these data, it can be concluded that bone substitute remodeling is supported by the AV loop. This corresponds with our previous study showing that an acceleration of vascularization leads to a faster bone substitute remodeling^12^. Contrary to what we expected, there was no significant difference in bone formation between the different scaffold groups with or without vascularization and growth factors, respectively. There was no significant difference in bone formation between groups 2–4 and the clinical gold standard (group 1), showing that harvesting autologous bone can be replaced by tissue engineering strategies. We made sure we created critical-size defects for optimal comparison of the different experimental groups. Defect sizes measured 3 cm and the periosteum was completely removed^40^; however, due to animal welfare and in accordance with the principles of the 3Rs (Replacement, Reduction, and Refinement), we did not include a group without intervention in order to prove the critical size. BMP-2 and VEGF-A were used in rather low concentrations (BMP-2: 135.95 µg, VEGF-A: 108.75 µg) compared to other studies in which BMP-2 was applied in the mg range. For example, Schuckert et al. used a concentration of 1.2 mg for mandibular defect reconstruction^41^, Carter et al. applied 1.5 mg/ml for mandibular defects^42^ and Wiltfang et al. used 12 mg BMP-2 for reconstructing a discontinuity in the mandible^20^. Using a higher concentration of BMP-2 and VEGF-A would probably enhance bone formation and vascularization within our setting and should be taken into account for further studies. Further, the lack of any difference in bone formation within our experimental groups could be due to the quite small sample size of *n* = 4 and *n* = 3, respectively. It is always possible for one animal to be included with impaired bone regeneration capacities, as shown in the study of Rozen et al.^33^, which would have a major influence due to the small sample size.

We consider the small sample size in this study as a major limitation. It was the aim of this study to provide a preclinical study for demonstrating this successful technique in a large animal model. Due to animal welfare and the high effort of large animal experiments, we decided to keep the group size rather small. Missing CT analysis and CTA of some animals as described in the results section is a further limitation of this study. In addition, CTA and micro-CT were not performed in the control group. In groups 2–4, bone area and vascularization could be easily quantified within the camber. However, since no chamber was implanted in the control group, it was rather difficult to determine the exact location of the measurement. We, therefore, decided to not include this group in this analysis since the objective assessment was not possible.

Pobloth et al. recently analyzed the effect of scaffolds and fixations techniques with different mechanical properties on bone healing. It could be shown that scaffolds with lower stiffness and a defect fixation exerting a higher mechanical force on the defect area lead to a better bone formation compared to defects that are shielded from stress^43^. Schreivogel et al. could further show that cyclic mechanical compression induces osteogenic differentiation of MSC^44^. The titanium chamber in our study allows an optimal stabilization of the defect, but at the same time leads to a rather low mechanical load in the defect area. Stabilization with an internal nail with load-bearing could most probably shorten the time until defect closure. We do not recommend using external fixation in our model due to lower load-bearing and higher risk of infections. It would be highly interesting to test different fixation techniques in combination with mechanical analysis and the effect on MSC and bone healing in future studies.

We consider this study as highly innovative since this technique can be easily translated into a clinical routine without significant effort. Our custom-made chamber-plate-fixation technique proved suitable for long-time defect stabilization and could easily be applied in the clinical setting. The chamber dimension can be adjusted individually to the patient’s requirement to engineer bone tissue that fits precisely into the defect site. Working with the clinically approved bone substitute material NanoBone^®^ overcomes regulatory obstacles. Further, this simple approach requires not more than microsurgical experience and the possibility to cultivate autologous cells for transplantation which opens up enormous opportunities for patients suffering from large-scale bone defects.

Using different components from the reconstructive armamentarium we were able to engineer customizable vascularized transplantable bone within one living organism. With this translational approach, we performed the proof of principle of load-bearing transplantable tissue-engineered bone in clinically relevant dimensions and paved the way towards translation into clinical application. In future studies, it would be of great interest to test the long-term effect and mechanical stability of this approach compared to the current gold standard. Replacing the gold standard of vascularized autologous bone in clinical routine would be a significant step towards individualized treatment concepts. Personalized medicine for the reconstruction of critical-size bone defects would lead to a reduction of side effects and faster integration back into daily social and working life.

## Methods

### Animals

For the present study, 17 female merino land sheep with a body weight of 30–40 kg were used. Experiments were approved by the Government of Unterfranken, Bavaria, Germany (Az. 54-2532.1-44/11; 55.2-2532-2-465). The animals were housed in an animal care facility as previously described^45^.

The animals were randomly divided into four groups: group 1 (*n* = 5), control group, implantation of autologous bone; group 2 (*n* = 4) implantation of bone substitute, MSC and EPC, growth factors BMP-2 and VEGF-A; group 3 (*n* = 4) implantation of bone substitute, MSC and EPC, growth factors BMP-2 and VEGF-A, AV loop; group 4 (*n* = 4) implantation of bone substitute, MSC and EPC, AV loop.

### Isolation and characterization of endothelial progenitor cells and mesenchymal stem cells

EPC were isolated from sheep whole blood using density gradient centrifugation. Up to 100 ml blood was taken from the external jugular vein and anticoagulated with heparin (60 IU/ml; Heparin-Natrium-25000-ratiopharm^®^, Ratiopharm GmbH, Ulm, Germany). In 50 ml centrifugal tubes, 10 ml blood was carefully pipetted on top of 10 ml Histopaque^®^−1.077 (Sigma-Aldrich, St. Louis, MO, USA) and centrifuged (20 min, 800x*g*, room temperature (RT), deceleration (dec) 0). The opaque interlayer was pipetted into a new centrifugal tube, washed three times with DMEM (Dulbecco’s Modified Eagle’s Medium; Sigma-Aldrich) and centrifuged (10 min, 300x*g*, 4 °C). Cells were resuspended in Endothelial Cell Growth Medium^TM^−2 (EGM^TM^−2; Lonza Group Ltd, Basel, Switzerland), containing the SingleQuots^TM^ Supplement Pack with heparin, gentamicin sulfate-amphotericin-1000, human epidermal growth factor (hEGF), ascorbic acid, arginine 3-insulin-like growth factor-1 (R3-IGF-1), vascular endothelial growth factor (VEGF), human fibroblast growth factor-basic (hFGF-B), hydrocortisone), and further 5% fetal calf serum superior (FCS sup; Biochrom AG, Hamburg, Germany). EPC were seeded in gelatin-coated (Cell Biologics Inc., Chicago, IL, USA) cell culture flasks and incubated at 37 °C, 5% CO_2_. The medium was changed every 2 to 3 days and cells were split at 90% confluence.

For characterization, EPC of one sheep were incubated with 10 µg/ml DiI AcLDL (low-density lipoprotein from human plasma, acetylated, DiI complex; Invitrogen™ Molecular Probes™ Thermo Fisher Scientific Inc., Waltham, MA, USA) for 4 h at 37 °C, fixated with 2% paraformaldehyde (PFA) (Sigma Aldrich), washed and incubated with 10 µg/ml FITC-conjugated lectin (Ulex europaeus, Sigma-Aldrich) for 60 min. Counterstaining was performed with DAPI (1 μg/ml, 10 min; Life Technologies, Carlsbad, CA, USA). For further characterization, PFA-fixated EPC were incubated for 1 h with an anti-CD34 rabbit monoclonal antibody (6.7 µg/ml, clone EP373Y; Abcam, Cambridge, UK). As a secondary antibody, goat anti-rabbit IgG (H+L) Alexa Fluor^®^ 488 conjugate was used (1 h, 10 µg/ml). Counterstaining was performed with DAPI as described above.

For the sprouting assay, cell spheroids were prepared using the hanging drop technique. Cells were suspended in EGM-2 medium supplemented with 5% FCS sup and 0.24% methocel^®^ (Sigma-Aldrich). Drops of 25 µl containing 750 EPC were pipetted on a petri dish and cultivated for 48 h at 37 °C upside down. Spheroids were embedded in fibrin (10 mg/ml, Baxter International Inc., Deerfield, IL, US) and incubated for 48 h.

For mRNA expression analysis, RNA of EPC and MSC of three sheep were isolated using an RNeasy Mini Kit with the corresponding QIAshredder Homogenizer (Qiagen, Hilden, Germany) and reverse-transcribed into cDNA using a QuantiTect Reverse Transcription Kit with a DNase I incubation (Qiagen). SsoAdvanced Universal SYBR Green Supermix (Bio-Rad Laboratories, Hercules, CA, USA) was used with a Light Cycler (Bio-Rad CFX96) for quantitative real-time PCR. All kits were used according to the manufacturer’s protocols. Samples were tested in triplicate. As housekeeping gene glyceraldehyde 3-phosphate dehydrogenase (GAPDH) was used. Data analysis was performed using the 2^-ΔΔCT^ method. Supplementary Table 1 specifies all primers used for real-time PCR.

MSC were isolated from the bone marrow of sheep using density gradient centrifugation as described previously^45^. Sheep were anesthetized with midazolam (0.5–1 mg/kg intramuscular (i.m.); Ratiopharm GmbH), and ketamine (5–10 mg/kg i.m., Ketavet^®^; Zoetis Inc., Parsippany, NJ, USA) and intubated. After shaving, disinfection, and local anesthesia (prilocaine hydrochloride, Xylonest^®^ 1%; AstraZeneca, London, UK), a small incision was made above the spina iliaca dorsalis cranialis (human anatomical correlate: posterior superior iliac spine) of the iliac crest. With an 11-gauge needle 20–30 ml bone marrow were aspirated into heparin-coated syringes (600 IE/ml). A 50 ml LeucoSEP^TM^ tube was filled with 16 ml Lymphoprep^TM^ and centrifuged (1 min, 218x*g*). Bone marrow was diluted 1:4 with phosphate-buffered saline (PBS, Biochrom AG, Berlin, Germany), filtered (100 µm), and directly pipetted on top of the filter of the LeucoSEP^TM^ tube and centrifuged (12 min, 1000x*g*, dec = 1). The opaque interlayer on top of the filter was washed with PBS, filtered (70 µm), and centrifuged (10 min, 314x*g*). Cells were resuspended in DMEM 4.5 g/l glucose (Sigma-Aldrich) supplemented with 10% FCS sup and 1% penicillin-streptomycin (all from Biochrom AG) and incubated at 37 °C, 5% CO_2_. The medium was changed every 2 to 3 days and cells were split at 90% confluence.

For characterization, MSC of three sheep were isolated according to the same protocol as for the implantation procedure and differentiated into adipogenic, chondrogenic, and osteogenic lineage. For the adipogenic differentiation, MSC were cultivated in a differentiation medium consisting of MSC adipogenic differentiation basal medium, supplemented with FCS, glutamine, insulin, 3-isobutyl-1-methyl-xanthine (IBMX), dexamethasone, rosiglitazone, and penicillin-streptomycin; for chondrogenic differentiation MSC were cultivated in MSC chondrogenic differentiation basal medium, supplemented with FCS, dexamethasone, ascorbate, sodium selenite (ITS), proline, TGF-β3, and penicillin-streptomycin; for osteogenic differentiation MSC were cultivated in MSC osteogenic differentiation basal medium, supplemented with FCS, glutamine, ascorbate, β-glycerophosphate, dexamethasone, and penicillin-streptomycin according to the manufacturer’s recommendation (all from Pelobiotech GmbH, Planegg, Germany). Successful differentiation was proved by staining the cells with Oil Red O, Alcian blue, and Alizarin red solution, respectively.

### Implantation chamber

**Fig. 6:**
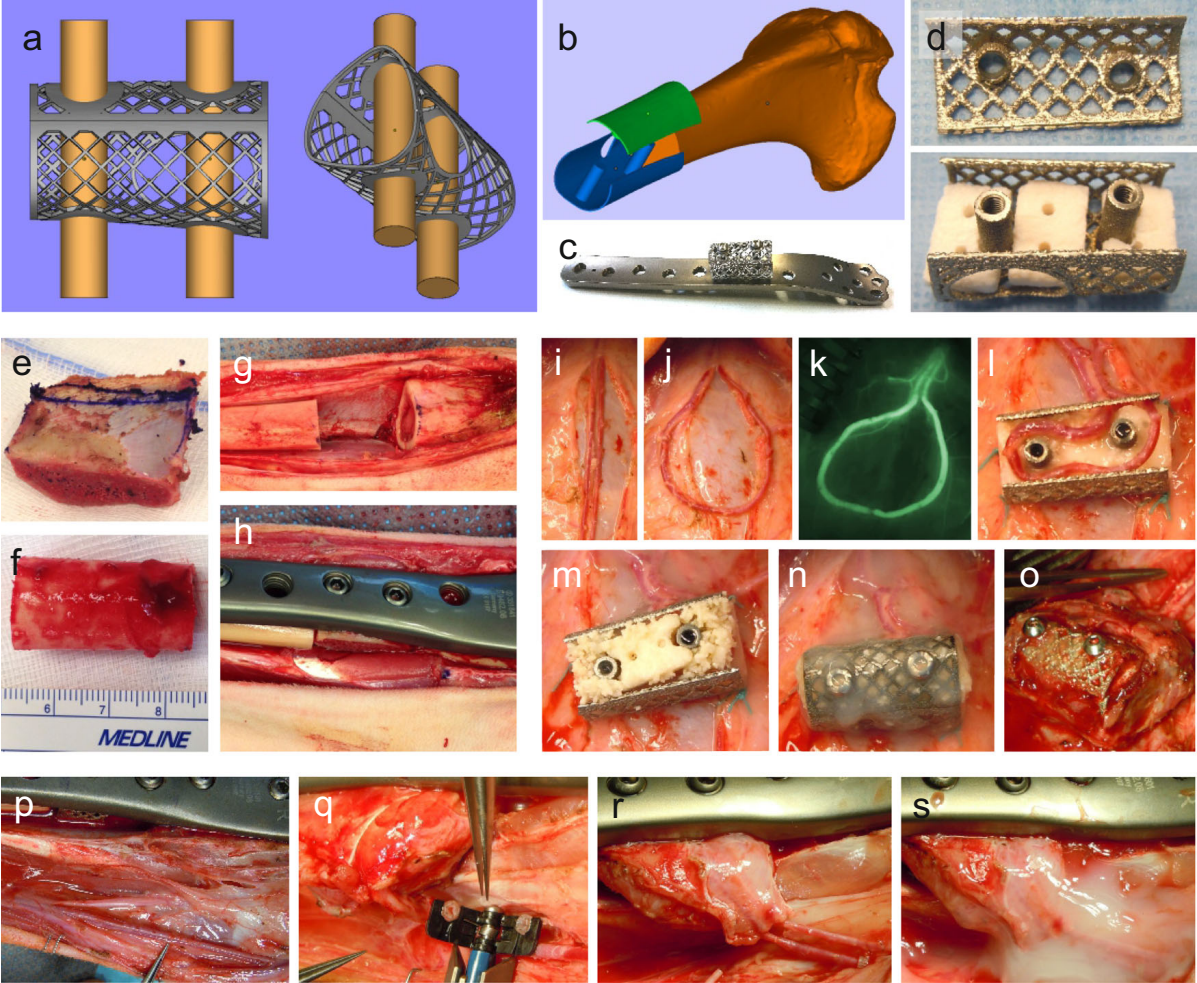
Surgery. Custom-made perforated titanium chamber for implantation into the sheep tibia defect. **a**, **b** Schema with bottom and lid. **c** Two connecting nuts for stable connection of the lid with the bottom of the chamber and for fixation at the osteosynthesis plate. **d** Chamber half-filled with the bone substitute NanoBone^®^ block. For implantation, a fourth block was placed on top of the middle bone block and gaps were completely filled with NanoBone^®^ granulate. **e** Part of the iliac crest after harvesting. **f**, **g** A tibia defect of 3 cm was created. **h** Iliac crest implanted in the tibia defect before fixation with the plate. **i** Medial thigh aspect: After dissection of the subcutaneous fatty layer the translucent superficial fascia cruris was incised and the vascular axis of the saphenous artery, vein, and nerve exposed. **j** AV loop was created microsurgically by micro-anastomosis with 9-0 Nylon interrupted sutures. **k** Perfusion of the AV loop was checked by indocyanine green (ICG) fluorescence imaging. **l** Half of the chamber was prefilled with a bone substitute, MSC and EPC and growth factors according to the experimental groups, and the AV loop was placed within the custom-made titanium chamber. **m** The chamber was completely filled with the bone substitute. **n** The lid was closed, the chamber was fixed in the groin and embedded in fibrin. **o** Tissue-engineered bone in the titanium chamber after 4 weeks of prevascularization before transplantation. **p** The vascular axes of the saphenous artery and vein at the bone defect site were exposed before transplantation of the tissue-engineered bone. **q** During vessel preparation the bone defect was stabilized with the bone defect plate with a place holder (empty chamber). Vein anastomosis was performed by a coupler system. **r** Tissue-engineered bone is in place in the tibia defect and fixed with the plate; artery and vein can be visualized. **s** Pedicle vessels are embedded in fibrin matrix for stabilization reasons.

The chamber was produced by selective electron beam melting of titanium powder^46^. A special perforated implantation chamber made of titanium was tailored to meet the specific requirements of the sheep tibia defect site (Fig. 6a, b). The length of the chamber was 30.0 mm with a diameter at the proximal end of 20.0 mm and at the distal end of 17.2 mm. The opening for the loop vessel had a diameter of 15.0 mm and a height of 10.0 mm. Two connecting nuts with a diameter of 6.0 mm served for stable connection of the lid with the chamber and further for fixation of the chamber at the osteosynthesis plate (Fig. 6c).

### Preparation of the bone substitute for implantation

In experimental groups with cell implantations (groups 2–4), MSC were fluorescent-labeled with CellTracker™ CM-DiI (Thermo Fisher) prior to implantation. The cell pellet was incubated with 5 µg/ml CellTracker™ CM-DiI in a two-step process (5 min, 37 °C, and 15 min, 4 °C). After washing the cells with PBS, 10 × 10^6^ MSC and 20 × 10^6^ EPC were resuspended in 3 ml DMEM. In all groups, autologous MSC and EPC were implanted. In experimental groups including growth factors (groups 2 and 3), the cell suspension was supplemented with a total dose of 135.95 µg BMP-2 and 108.75 µg VEGF-A per scaffold (both from PeproTech Germany, Hamburg, Germany).

As bone replacement material, the clinically approved nanocrystalline NanoBone^®^ block with a size of 0.5 cm × 1.5 cm × 1.0 cm (Artoss GmbH, Rostock, Germany) and NanoBone^®^ granulate (1.0 mm × 2.0 mm) were used. Four blocks and 2.4 ml granulate were mixed with the suspension of cells and growth factors, and incubated under vacuum for 40 s for optimal soaking of the bone substitute. Three blocks were placed side by side onto the bottom of the chamber and one block was placed on top of the middle block in between the connecting nuts (Fig. 6d). In groups 3 and 4 the loop vessel was placed around the two connecting nuts in the chamber, and completely filled with NanoBone^®^ granulate (1.0 mm × 2.0 mm). The chamber was closed with the lid by using two screws that were screwed into the connecting nuts.

### Surgery

Prior to surgery, animals were deprived of food for 24 h to limit regurgitation. Anesthesia was induced with midazolam and ketamine as described above. Sheep were intubated and connected to the respirator (Drägerwerk AG & Co. KGaA, Lübeck, Germany) for maintaining anesthesia by inhalation of a gas mixture of 1–2% isoflurane (Baxter GmbH Deutschland, Unterschleißheim, Germany), air, and oxygen. The animals were subjected to controlled artificial respiration with weight-adapted breath volume to keep exhaled CO_2_ between 35 and 45 mmHg. To reduce abdominal bloating a stomach tube was inserted into the esophagus. Intraoperative volume loss was adjusted by weight-adapted intravenous (i.v.) infusion of crystalloids (4 ml/kg/h, Fresenius Kabi AG, Bad Homburg, Germany). A catheter was introduced into the bladder prior to surgery. Pre- and intraoperatively, carprofen (4 mg/kg subcutaneously (s.c.), Rimadyl^®^; Pfizer, Berlin, Germany), and fentanyl (i.v. bolus 5 µg/kg and 5–10 µg/kg/h, Fentanyl-Janssen 0.5 mg, Janssen-Cilag GmbH, Neuss, Germany) were administered to ensure analgesia. Antibiotics (Cefquinom 1–1.25 mg/kg i.m.; Cobactan^®^, Intervet Deutschland GmbH, Unterschleißheim, Germany) were given prior to surgery.

All operations were performed by the same microsurgeon using a surgical microscope (Leica Microsystems, Wetzlar, Germany). Vessel preparation was performed under a binocular at 3.5x magnification.

### Harvesting of a nonvascularized iliac crest and tibia defect (group 1)

After shaving and disinfection, an incision was made above the tuber coxae/anterior edge to the crista iliaca (human anatomical correlate: anterior superior iliac spine) of the iliac crest. Preparation was performed down to the periost and incised. A 3 cm bone graft was harvested from the iliac crest with a saw (Fig. 6e). Hemostasis, periost suture, and wound closure using Prolene 2-0 (Ethicon Inc., Somerville, NJ, USA) were performed.

The surgical site at the tibia was shaved, prepped, and draped for sterility. A longitudinal skin incision was made over the tibia defect site after X-ray control. After dissection of the thin subcutaneous fatty layer, the translucent superficial fascia cruris was exposed and incised. The periost was incised and completely removed at the defect site. Under X-ray control, the tibia defect was planned in the middle aspect of the tibia. The plate was fixed with k-wires under X-ray control and the 3 cm bone defect was created with a saw (Fig. 6f, g). The iliac bone graft was placed in the defect site. The tibia and the bone graft were fixated by the plate and screws (Königsee Implantate, distal femur plate set, titanium, Allendorf, Germany) under X-ray control (Fig. 6h). Hemostasis was assured, a suction drain was inserted, penetrating the sheep’s skin remote to the skin incision, and the wound was closed using Prolene 2-0.

### Tibia defect without AV loop (group 2)

The titanium chamber was filled with the bone substitute, cells, and growth factors and fixed to the plate with two titanium screws. After the creation of the tibia defect in the same way as group 1, the plate with the chamber was placed onto the tibia and the plate was fixed with titanium screws. The chamber was positioned inside the defect with cortical contact to the tibia. Hemostasis, suction drain, and wound closure were performed as described above.

### AV loop surgery (groups 3 and 4)

The surgical site was shaved, prepped, and draped for sterility. A longitudinal 12 cm skin incision was made reaching proximally from the groin down to the medial thigh aspect. After dissection of the thin subcutaneous fatty layer the translucent superficial fascia cruris was exposed and the vascular axis of the saphenous artery, vein, and nerve became visible. The superficial fascia was cut and the vessels were dissected microsurgically, starting from the origin in the groin distally over a length of 12 cm (Fig. 6i). Side branches were coagulated using micro-bipolar forceps. After proximal clamping and distal ligation of the artery and vein, micro-anastomosis of the proximal ends of the saphenous artery and vein was performed using 9-0 Nylon interrupted sutures (Ethicon Inc.) (Fig. 6j). 100 IU/kg heparin was given i.v. Perfusion of the AV loop was checked by indocyanine green (ICG) fluorescence imaging (Fig. 6k). About 1.5 mg/kg (VERDYE 5 mg/ml, Diagnostic Green GmbH, Aschheim, Germany) was injected i.v. and simultaneously perfusion of the vascular system was visualized with an IC-Flow™ Imaging System (Diagnostic Green GmbH). The AV loop was placed into the custom-made sterile isolation chamber, which was half-filled with the bone substitute (Fig. 6l), MSC and EPC as well as growth factors in group 3 as described above. A fibrin matrix was prepared by mixing 6 ml of fibrinogen (20 mg/ml) together with 6 ml of thrombin (500 IE/ml) using the DUPLOJECT-Two Syringe System (Baxter International Inc.), which was applied to the AV loop vessels for fixation and embedding. The chamber was completely filled with the bone substitute (Fig. 6m). The lid was closed and the chamber fixed in the groin and embedded in fibrin (Fig. 6n). Hemostasis was assured, a closed suction drain was inserted, penetrating the sheep’s skin remote to the skin incision, and the wound was closed using Prolene 2-0 (Ethicon Inc.). The prevascularization period was 4 weeks.

### Transplantation of the tissue-engineered axially vascularized bone graft

The surgical site was shaved, prepped, and draped for sterility. A longitudinal 15 cm skin incision on the medial thigh aspect was made and the tissue-engineered bone graft within the titanium chamber was exposed. The vessels of the AV loop were microsurgically prepared over a length of 5–7 cm proximal to the chamber (Fig. 6o). 100 IU/kg heparin was given i.v. After distal clamping and proximal ligation of the artery and vein, the construct was explanted. The artery was cannulated and continuously perfused by infusion (gravity) with 1000 ml crystalloids with 100 IU/ml heparin and 0.05 mg/ml papaverine hydrochloride (Paveron^®^ N, Linden Arzneimittel Vertrieb GmbH, Heuchelheim, Germany) and stored at 4 °C until transplantation. Afterward, the sheep was turned onto the other side and the surgical site was shaved, prepped, and draped for sterility. A longitudinal 15 cm skin incision on the medial thigh aspect was planned, depending on the tibia defect site. After dissection of the thin subcutaneous fatty layer, the translucent superficial fascia cruris was exposed and the vascular axis of the saphenous artery, vein, and nerve became visible (Fig. 6p). The superficial fascia was cut and the vessels were dissected microsurgically. For successful anastomosis, the vessels were dissected until an adequate diameter was reached (artery/vein 1–2 mm). Side branches were coagulated using micro-bipolar forceps. The tibia defect was created as described above and the titanium chamber with the tissue-engineered bone was placed into the defect and fixed to the plate and the plate fixed to the tibia with screws. After proximal clamping and distal ligation of the artery and vein, micro-anastomosis of the proximal ends of the saphenous artery and vein with the AV loop vessels of the tissue-engineered construct was performed using 9-0 Nylon (Ethicon Inc., Norderstedt, Germany) interrupted sutures for the artery and the Microvascular Anastomotic COUPLER System™ (TapMed Medizintechnik, Habichtswald-Ehlen, Germany) for the vein and subsequently embedded in fibrin (Fig. 6q–s). 100 IU/kg heparin was given i.v. as a bolus. Blood flow was monitored by a Doppler probe. Hemostasis was assured, a closed suction drain was inserted, penetrating the sheep’s skin remote to the skin incision, and the wound was closed using Prolene 2-0 (Ethicon Inc.). Immobilization and stabilization of the bone were ensured using a cast for the whole implantation period.

### Postoperative care and monitoring of successful transplantation

Buprenorphine (0.001–0.01 mg/kg s. c., Buprenovet, Animalcare Ltd, York, Great Britain) was administered to the operated sheep before awakening and afterward every 4–8 h for about 5 days. Carprofen was given once daily for the next 3 days, and cefquinome was given once daily for about 7–10 days. To prevent thrombosis of the loop vessels, sodium enoxaparin (2.5 mg/kg s. c., Clexane multidose^®^, Sanofi-Aventis, Paris, France) was given at the time of vessel anastomosis in a concentration of 5 mg/kg and afterward twice daily in a concentration of 2.5 mg/kg. In addition, sheep received 300 mg clopidogrel per os (p.o.) (Plavix^®^, Sanofi-Aventis) directly after awakening and afterward in a concentration of 150 mg twice daily. Anticoagulation was performed according to a previous study^47^.

An X-ray of the bone defect was performed 1, 2, 4, 6, 8, 10, and 12 weeks postoperatively (63 kV, 8.0 mAs). Once a week Doppler sonography was performed in groups 3–4 to ensure patency of the loop vessels. Simultaneously, operation wounds were checked and the immobilization cast was renewed.

### In vivo computed tomography and angiography

At time points 4 and 12 weeks after bone defect surgery, computed tomography (CT) and angiography (CTA) were performed in vivo for quantification of bone density and microcirculation in the defect area. Sheep were anesthetized as described above using midazolam and ketamine. Infusion with weight-adapted crystalloids (4 ml/kg/h, Fresenius Kabi AG) was given throughout the procedure. In vivo imaging by CT was performed at week 4 and week 12 after surgery. Morphological CT imaging was performed (CT scanner SOMATOM Force, Siemens Healthineers, Erlangen, Germany) with the following setting parameters: Dual Energy, Isocenter, Bone Window, Eff mAs 450, a voltage of 100 KV and Sn150 KV, craniocaudal and sagittal/coronal direction, layer 0.6 mm, increment 0.4 mm, Acq. 192 × 0.6 mm, rotation time 0.5 s, Pitch 0.5, Br 64, FoV 264 mm, center X 93 mm, center Y 22 mm, ADMIRE intensity 2.

The Angio CT imaging “DynMulti4D” was performed with the following setting parameters: 200 mAs, voltage of 70 KV, a layer of 3 mm, caudocranial direction, Acq. 192 × 0.6 mm, increment of 3 mm, rotation time 0.25 s, Br 59, FoV 190 mm. The contrast agent used for this measurement was Ultravist 370.

For image analyses, 3D multiplanar reconstructions were generated using the DICOM viewer aycan OsiriX, and the density of regions of interest (ROI) was measured in 11 different axial planes distributed over the area of the bone defect. The applied ROI area was the same in each animal. The 3D VR reconstructions (VTK) were generated using the DICOM viewer aycan OsiriX Pro.

### Explantation and micro-CT

For explantation, sheep were anesthetized as described for the AV loop surgery. Euthanasia was performed in deep anesthesia using T61^®^ (5–10 ml/50 kg i.v., Intervet International GmbH, Unterschleißheim, Germany). The operated leg was amputated and the femoral artery was immediately flushed with saline supplemented with 100 IU/ml heparin and 0.05 mg/ml papaverine hydrochloride until all vessels were flushed through. The femoral artery was perfused with the contrast agent Microfil^®^ (MV-122, Flow Tech Inc., Carver, MA, US). After the curing process at 4 °C for 24 h, the bone defect area with adjacent parts of the tibia (about 1 cm proximal and distal to the borders of the defect) was removed by using a saw. The explants were formalin fixated (4% buffered formaldehyde, 48 h, 4 °C) and decalcified by EDTA (Sigma-Aldrich) in an ultrasonic bath (Sonocool^®^ 255 Bandelin GmbH & Co. KG, Berlin, Germany). Afterward, micro-CT scans were performed. The ex vivo CT imaging was performed using a dedicated preclinical scanner (Inveon, Siemens Healthineers) at a tube voltage of 80 kV and a tube current of 500 μA. CT images were acquired with a resolution of 48.97 μm, and with the following setting parameters: step-and-shoot acquisition, full rotation, settle time 2000 ms, 360 projections, exposure time of 1100 ms, binning 2 × 2, charge-coupled device (CCD) size 2368 × 2048 px, FoV 58 × 50 mm, 40 min scan time. The DICOM viewer aycan OsiriX Pro with the Segmentation Program (Chimaera GmbH) was used for image analyses and volume measurements.

3D Maximum Intensity Projections were generated using the freeware DICOM viewer Horos Version 3.2.0.

### Histology

For histomorphometric analyses and immunohistochemical stainings, the explanted constructs were divided into four parts. First, they were cut along their longitudinal axis, and second, transverse to their longitudinal axis directly in the middle of the defect area. After dehydration, 3-µm-paraffin-slides were prepared for histology (MICROM International GmbH, Walldorf, Germany).

### Histomorphometric analyses and immunohistochemical staining

For quantification of newly formed bone and bone substitute area, slides were stained with hematoxylin and eosin (HE). From each of the four parts of one explanted construct, cross sections were obtained from two standardized planes. The complete slide was automatically scanned in 25X magnification and single images were assembled into one overview picture (Olympus IX83, CellSens software; Olympus). Semi-automatic analysis of a standardized ROI was performed using the Leica Application Suite V3 according to the user’s manual (interactive measurement of the Leica Application Suite, Leica Microsystems, Wetzlar, Germany). The ROI was defined as the area starting from the original border of the bone defect up to the middle of each construct. Typical coloring of the newly formed bone area was measured and referred to the total area of each slide. The remaining bone substitute was visible as a homogenous acellular structure. The area was measured and calculated with respect to the total area of the construct.

For quantification of vascularization, a von Willebrand factor (vWF) and alpha-smooth muscle actin (α-SMA) double immunohistochemical staining was performed using a Double Stain Polymer Kit (Zytomed Systems GmbH, Berlin, Germany). Slides were pretreated with 0.1% protease from Streptomyces griseus (Sigma-Aldrich) for 20 min and blocking steps were performed according to the manufacturer’s recommendation.

Slides were incubated with a mouse anti-smooth muscle actin antibody (1:80, clone 1A4; Zytomed Systems GmbH) for 1 h followed by incubation with a rabbit polyclonal vWF antibody (1:30; Biocare Medical, Concord, CA, USA) for 1.5 h. From each of the four parts of one construct cross sections were obtained from two standardized planes of paraffin-embedded constructs. Six standardized ROIs per slide were captured in 100X magnification. The number and area of vessels were calculated per ROI (ImageJ 1.53a, Open Source Software Rasband, W.S., ImageJ, US National Institutes of Health, Bethesda, Maryland, USA).

For visualization of bone formation, the Double Stain Polymer Kit (Zytomed Systems GmbH) was used with an anti-collagen type I polyclonal rabbit antibody (1:700, 1 h; Abcam) and an alkaline phosphatase (ALP) primary (tissue nonspecific) polyclonal rabbit antibody (1:100, 1 h; Genetex Inc., Irvine, CA, US). Pretreatment of slides was performed in a steamer for 5 min. Before the second antibody incubation, an elution step had to be performed due to the two primary antibodies having the same origin (10 min steaming in deionized water). For visualization of the matrix change of the bone substitute from an inorganic into an organic matrix, periodic acid Schiff (PAS) staining was used (PAS Staining Kit, Carl Roth GmbH & Co. KG, Karlsruhe, Germany). Tartrate-resistant acidic phosphatase (TRAP)-positive, osteoclast-like cells were detected with the histochemical TRAP staining as previously described^29^. Vessels were further stained with an anti-ovine CD31 antibody (1:70, 1 h; Anti-CD31/PECAM-1, Bio-Rad Laboratories Inc., Hercules, CA, USA) using a biotin-free tyramide signal amplification system (CSAII-System, Dako, Glostrup, Denmark).

### Statistical analyses

Data were expressed as mean ± standard deviation unless otherwise stated. Statistical analysis was performed using SPSS 20.0 for Windows (SPSS Inc., IL, USA). Due to the small sample size, results were interpreted using the nonparametric Kruskal–Wallis test and the Mann–Whitney *U*-test (two-sided, the asymptotic significance was used). The level of statistical significance was set to *p* < 0.05. All graphics were created with Origin 8.5. The brightness, contrast, and intensity of the depicted images were adapted for better perceptibility. Adaptions were made to the entire picture.

### Reporting Summary

Further information on research design is available in the Nature Research Reporting Summary linked to this article.

## Supplementary information

Supplementary Table 1

Reporting Summary

## Data Availability

The data that support the findings of this study are available from the corresponding author upon reasonable request.

## References

[1] Antonini A (2019). Bone defect management with vascularized fibular grafts in the treatment of grade III-IV osteomyelitis. Handchir. Mikrochir. Plast. Chir..

[2] Junnila J, Repo JP, Mustonen A, Tukiainen EJ (2015). Treatment of compound tibial fracture with free osteomuscular latissimus dorsi scapula flap. J. Reconstr. Microsurg..

[3] Repo JP, Barner-Rasmussen I, Roine RP, Sintonen H, Tukiainen E (2016). Role of free iliac crest flap in foot and ankle reconstruction. J. Reconstr. Microsurg..

[4] Forrestal DP, Klein TJ, Woodruff MA (2017). Challenges in engineering large customized bone constructs. Biotechnol. Bioeng..

[5] Shahabipour F (2020). Key components of engineering vascularized 3-dimensional bioprinted bone constructs. Transl. Res..

[6] Wang XY (2014). Engineering interconnected 3D vascular networks in hydrogels using molded sodium alginate lattice as the sacrificial template. Lab Chip.

[7] Kerouredan O (2019). Micropatterning of endothelial cells to create a capillary-like network with defined architecture by laser-assisted bioprinting. J. Mater. Sci. Mater. Med..

[8] Wimmer RA (2019). Human blood vessel organoids as a model of diabetic vasculopathy. Nature.

[9] Warnke PH (2004). Growth and transplantation of a custom vascularised bone graft in a man. Lancet.

[10] Kokemueller H (2010). Prefabrication of vascularized bioartificial bone grafts in vivo for segmental mandibular reconstruction: experimental pilot study in sheep and first clinical application. Int J. Oral. Maxillofac. Surg..

[11] Weigand A, Horch RE, Boos AM, Beier JP, Arkudas A (2018). The arteriovenous loop: engineering of axially vascularized tissue. Eur. Surg. Res..

[12] Weigand A (2015). Acceleration of vascularized bone tissue-engineered constructs in a large animal model combining intrinsic and extrinsic vascularization. Tissue Eng. Part A.

[13] Boos AM (2014). Autologous serum improves bone formation in a primary stable silica-embedded nanohydroxyapatite bone substitute in combination with mesenchymal stem cells and rhBMP-2 in the sheep model. Int J. Nanomed..

[14] Boos AM (2011). Directly auto-transplanted mesenchymal stem cells induce bone formation in a ceramic bone substitute in an ectopic sheep model. J. Cell Mol. Med..

[15] Vacanti CA (2006). The history of tissue engineering. J. Cell Mol. Med.

[16] Rouwkema J, Khademhosseini A (2016). Vascularization and angiogenesis in tissue engineering: beyond creating static networks. Trends Biotechnol..

[17] Yamada Y (2013). Minimally invasive approach with tissue engineering for severe alveolar bone atrophy case. Int. J. Oral. Maxillofac. Surg..

[18] Melville JC (2020). Is reconstruction of large mandibular defects using bioengineering materials effective?. J. Oral. Maxillofac. Surg..

[19] Melville JC, Tursun R, Green JM, Marx RE (2017). Reconstruction of a post-traumatic maxillary ridge using a radial forearm free flap and immediate tissue engineering (Bone morphogenetic protein, bone marrow aspirate concentrate, and cortical-cancellous bone): case report. J. Oral. Maxillofac. Surg..

[20] Wiltfang J (2016). Man as a living bioreactor: prefabrication of a custom vascularized bone graft in the gastrocolic omentum. Tissue Eng. Part C. Methods.

[21] Naujokat H, Acil Y, Gulses A, Birkenfeld F, Wiltfang J (2018). Man as a living bioreactor: long-term histological aspects of a mandibular replacement engineered in the patient’s own body. Int. J. Oral. Maxillofac. Surg..

[22] Wang L (2010). Osteogenesis and angiogenesis of tissue-engineered bone constructed by prevascularized beta-tricalcium phosphate scaffold and mesenchymal stem cells. Biomaterials.

[23] Vidal L (2020). Regeneration of segmental defects in metatarsus of sheep with vascularized and customized 3D-printed calcium phosphate scaffolds. Sci. Rep..

[24] Horch RE, Beier JP, Kneser U, Arkudas A (2014). Successful human long-term application of in situ bone tissue engineering. J. Cell Mol. Med..

[25] Warnke PH (2006). Man as living bioreactor: fate of an exogenously prepared customized tissue-engineered mandible. Biomaterials.

[26] Zhang W (2014). VEGF and BMP-2 promote bone regeneration by facilitating bone marrow stem cell homing and differentiation. Eur. Cell Mater..

[27] Feng L (2013). Effects of vascular endothelial growth factor 165 on bone tissue engineering. PLoS ONE.

[28] Ramasamy SK (2016). Blood flow controls bone vascular function and osteogenesis. Nat. Commun..

[29] Boos AM (2013). Engineering axially vascularized bone in the sheep arteriovenous-loop model. J. Tissue Eng. Regenerative Med..

[30] Schlund M, Nicot R, Depeyre A, Alkasbi J, Ferri J (2019). Reconstruction of a large posttraumatic mandibular defect using bone tissue engineering with fresh-frozen humeral allograft seeded with autologous bone marrow aspirate and vascularized with a radial forearm flap. J. Craniofac. Surg..

[31] Ahn G, Lee JS, Yun WS, Shim JH, Lee UL (2018). Cleft alveolus reconstruction using a three-dimensional printed bioresorbable scaffold with human bone marrow cells. J. Craniofac. Surg..

[32] Tilley S (2006). Taking tissue-engineering principles into theater: augmentation of impacted allograft with human bone marrow stromal cells. Regen. Med..

[33] Rozen N (2009). Transplanted blood-derived endothelial progenitor cells (EPC) enhance bridging of sheep tibia critical size defects. Bone.

[34] Rehman J, Li J, Orschell CM, March KL (2003). Peripheral blood “endothelial progenitor cells” are derived from monocyte/macrophages and secrete angiogenic growth factors. Circulation.

[35] Tamari, T. et al. The paracrine role of endothelial cells in bone formation via CXCR4/SDF-1 pathway. *Cells*10.3390/cells9061325 (2020). 10.3390/cells9061325 PMC7349013 32466427

[36] Peng J (2019). Bone marrow mesenchymal stem cells and endothelial progenitor cells co-culture enhances large segment bone defect repair. J. Biomed. Nanotechnol..

[37] Sun K (2016). Combined transplantation of mesenchymal stem cells and endothelial progenitor cells for tissue engineering: a systematic review and meta-analysis. Stem Cell Res. Ther..

[38] Berner A (2015). Delayed minimally invasive injection of allogenic bone marrow stromal cell sheets regenerates large bone defects in an ovine preclinical animal model. Stem Cells Transl. Med..

[39] Miguel-Sanchez A, Vilaplana-Vivo J, Vilaplana-Vivo C, Vilaplana-Gomez JA, Camacho-Alonso F (2015). Accuracy of quantitative computed tomography bone mineral density measurements in mandibles: a cadaveric study. Clin. Implant Dent. Relat. Res..

[40] Sparks DS (2020). A preclinical large-animal model for the assessment of critical-size load-bearing bone defect reconstruction. Nat. Protoc..

[41] Schuckert KH, Jopp S, Teoh SH (2009). Mandibular defect reconstruction using three-dimensional polycaprolactone scaffold in combination with platelet-rich plasma and recombinant human bone morphogenetic protein-2: de novo synthesis of bone in a single case. Tissue Eng. Part A.

[42] Carter TG, Brar PS, Tolas A, Beirne OR (2008). Off-label use of recombinant human bone morphogenetic protein-2 (rhBMP-2) for reconstruction of mandibular bone defects in humans. J. Oral. Maxil Surg..

[43] Pobloth, A. M. et al. Mechanobiologically optimized 3D titanium-mesh scaffolds enhance bone regeneration in critical segmental defects in sheep. *Sci. Transl. Med*. 10.1126/scitranslmed.aam8828 (2018). 10.1126/scitranslmed.aam8828 29321260

[44] Schreivogel S, Kuchibhotla V, Knaus P, Duda GN, Petersen A (2019). Load-induced osteogenic differentiation of mesenchymal stromal cells is caused by mechano-regulated autocrine signaling. J. Tissue Eng. Regenerative Med..

[45] Beier JP (2010). Axial vascularization of a large volume calcium phosphate ceramic bone substitute in the sheep AV loop model. J. Tissue Eng. Regenerative Med..

[46] Pobel, C., Gotterbarm, M., Samfaß, V., Osmanlic, F. & Körner, C. *Proc. 6th International Conference on Additive Technologies* (eds Dietmar, D., Igor, D. & Michael, S.) 130–137 (Interesansa - zavod, 2016).

[47] Weigand A (2013). New aspects on efficient anticoagulation and antiplatelet strategies in sheep. BMC Vet. Res.

