# Supplementary material for: Personalized medicine for reconstruction of critical-size bone defects – a translational approach with customizable vascularized bone tissue
Source: NPJ Regen Med. 2021 Aug 19;6:49. doi: 10.1038/s41536-021-00158-8 (PMC8377075; doi:10.1038/s41536-021-00158-8)
Supplement: Supplementary file 1 — Supplementary Table 1 [file 41536_2021_158_MOESM1_ESM.pdf]

Supplementary Table 1. Primer sequences

gene	Forward 5‘-3‘	Reverse 5‘-3‘
C31	GCTGACACTCCTGCTCTGTT	GTTTTGCACCGTGTTTTGCG
CD34	TGAACCCTTTAGCCGCTCTG	CTGGGTCACCTGCAGAAGAG
GAPDH	TGACCCCTTCATTGACCTTC	GATCTCGCTCCTGGAAGATG
VEGFR2	TATCCAAGCGGCCAATGTGT	CCCTCTCTCCTCTTCCAGCT
VWF	GCTTACCCAGGTGTCAGTCC	GCTTTGTCCACGTGCACATT
