# Supplementary material for: Personalized medicine for reconstruction of critical-size bone defects – a translational approach with customizable vascularized bone tissue
Source: NPJ Regen Med. 2021 Aug 19;6:49. doi: 10.1038/s41536-021-00158-8 (PMC8377075; doi:10.1038/s41536-021-00158-8)
Supplement: Supplementary file 2 — Reporting Summary [file 41536_2021_158_MOESM2_ESM.pdf]

nature research

Corresponding author(s): Annika Kengelbach-Weigand

Last updated by author(s): May 31, 2021

Reporting Summary

Nature Research wishes to improve the reproducibility of the work that we publish. This form provides structure for consistency and transparency in reporting. For further information on Nature Research policies, see our Editorial Policies and the Editorial Policy Checklist.

Statistics

For all statistical analyses, confirm that the following items are present in the figure legend, table legend, main text, or Methods section.

n/a	Confirmed	
☐	☒	The exact sample size (*n*) for each experimental group/condition, given as a discrete number and unit of measurement
☐	☒	A statement on whether measurements were taken from distinct samples or whether the same sample was measured repeatedly
☐	☒	The statistical test(s) used AND whether they are one- or two-sided<br>*Only common tests should be described solely by name; describe more complex techniques in the Methods section.*
☒	☐	A description of all covariates tested
☒	☐	A description of any assumptions or corrections, such as tests of normality and adjustment for multiple comparisons
☒	☐	A full description of the statistical parameters including central tendency (e.g. means) or other basic estimates (e.g. regression coefficient) AND variation (e.g. standard deviation) or associated estimates of uncertainty (e.g. confidence intervals)
☐	☒	For null hypothesis testing, the test statistic (e.g. *F*, *t*, *r*) with confidence intervals, effect sizes, degrees of freedom and *P* value noted<br>*Give P values as exact values whenever suitable.*
☒	☐	For Bayesian analysis, information on the choice of priors and Markov chain Monte Carlo settings
☒	☐	For hierarchical and complex designs, identification of the appropriate level for tests and full reporting of outcomes
☒	☐	Estimates of effect sizes (e.g. Cohen's *d*, Pearson's *r*), indicating how they were calculated

Our web collection on statistics for biologists contains articles on many of the points above.

Software and code

Policy information about availability of computer code

Data collection	n/a
Data analysis	n/a

For manuscripts utilizing custom algorithms or software that are central to the research but not yet described in published literature, software must be made available to editors and reviewers. We strongly encourage code deposition in a community repository (e.g. GitHub). See the Nature Research guidelines for submitting code & software for further information.

Data

Policy information about availability of data

All manuscripts must include a data availability statement. This statement should provide the following information, where applicable:

- Accession codes, unique identifiers, or web links for publicly available datasets
- A list of figures that have associated raw data
- A description of any restrictions on data availability

The data that support the findings of this study are available from the corresponding author upon reasonable request.

Field-specific reporting

Please select the one below that is the best fit for your research. If you are not sure, read the appropriate sections before making your selection.

☒ Life sciences ☐ Behavioural & social sciences ☐ Ecological, evolutionary & environmental sciences

For a reference copy of the document with all sections, see nature.com/documents/nr-reporting-summary-flat.pdf

Life sciences study design

All studies must disclose on these points even when the disclosure is negative.

Sample size	Sample size: n= 4 or 5 were used. No sample-size calculation was performed before, because we performed a proof-of-principle study. No data were available for sample-size calculation, because this experiment was never done before. Due to animal welfare we did not include more animals, because suitability of this method could be shown with even this small sample size.
Data exclusions	One animal was excluded from this study because of low cell yield, this is stated in the manuscript.
Replication	Replication was not possible due to animal welfare. For all histological analyses we used multiple slides as described in the manuscript.
Randomization	Animals were randomly allocated into the experimental groups.
Blinding	Blinding was not possible, because investigators involved in data analyses were the same investigators who prepared the samples and performed imaging measurements. We assume that blinding would have no effect on our results.

Reporting for specific materials, systems and methods

We require information from authors about some types of materials, experimental systems and methods used in many studies. Here, indicate whether each material, system or method listed is relevant to your study. If you are not sure if a list item applies to your research, read the appropriate section before selecting a response.

Materials & experimental systems

n/a	Involved in the study	
☐	☒	Antibodies
☒	☐	Eukaryotic cell lines
☒	☐	Palaeontology and archaeology
☐	☒	Animals and other organisms
☒	☐	Human research participants
☐	☐	Clinical data
☒	☐	Dual use research of concern

Methods

n/a	Involved in the study	
☒	☐	ChIP-seq
☒	☐	Flow cytometry
☒	☐	MRI-based neuroimaging

Antibodies

Antibodies used	CD34 staining: anti-CD34 rabbit monoclonal antibody (6.7 µg/ml, clone EP373Y; cat. Number ab81289, Abcam, Cambridge, UK).<br>vWF staining: rabbit polyclonal vWF antibody (1:30; clone: N/A (not provided by the manufacturer, cat. Number CP 039 A, B, Biocare Medical, Concord, CA, USA) 1.5 h.<br>alpha smooth muscle actin staining: mouse anti-smooth muscle actin antibody (1:80, clone 1A4; cat. Number MSK030 Zytomed Systems GmbH) for 1 h<br>collagen type I staining: anti-collagen type I polyclonal rabbit antibody (1:700, 1 h; cat. Number ab34710; Abcam)<br>ALP staining: alkaline phosphatase (ALP) primary (tissue non-specific) polyclonal rabbit antibody (1:100, 1 h; cat. Number GTX100817, LOT 40051, Genetex Inc., Irvine, CA, US)<br>CD31 staining: anti-ovine CD31 antibody (1:70, 1 h; Anti-CD31/PECAM-1, Cat. Number MCA1097GA, clone CO.3E1D4, MorphoSys, Kidlington, Oxford, UK)
Validation	• Validation CD34: according to the manufacturer reacts with sheep and suitable for ICC/IF<br>• Validation vWF: according to the manufacturer reacts with human, mouse, rat and suitable for IHC-P. Validation was performed with sheep blood vessel tissue which stained positively (also visible in the publication. See Fig. 6)<br>• Validation aSMA: according to the manufacturer reacts with human, mouse, rat (others not tested) and suitable for IHC-P. Validation was performed with sheep blood vessel tissue which stained positively (also visible in the publication. See Fig. 6)<br>• Validation collagen type I: according to the manufacturer reacts with Mouse, Sheep, Cow, Human, Pig, Common marmoset and suitable for IHC-P.<br>• Validation ALP: according to the manufacturer reacts Human, Mouse, Sheep and suitable for IHC-P.<br>• Validation CD31: according to the manufacturer reacts with sheep, IHC-P not tested. Validation was performed with sheep blood vessel tissue which stained positively (also visible in the publication. See Fig. 6)

Animals and other organisms

Policy information about studies involving animals; ARRIVE guidelines recommended for reporting animal research

Laboratory animals	female merino land sheep with a body weight of 30-40 kg, aged about 6 months
Wild animals	n/a
Field-collected samples	n/a
Ethics oversight	Experiments were approved by the Government of Unterfranken, Bavaria, Germany (Az. 54-2532.1-44/11; 55.2-2532-2-465)

Note that full information on the approval of the study protocol must also be provided in the manuscript.

Clinical data

Policy information about clinical studies

All manuscripts should comply with the ICMJE guidelines for publication of clinical research and a completed CONSORT checklist must be included with all submissions.

Clinical trial registration	n/a
Study protocol	n/a
Data collection	n/a
Outcomes	n/a
